# Trends and projections in sexually transmitted infections in people aged 45 years and older in England: analysis of national surveillance data

**DOI:** 10.1177/17579139221106348

**Published:** 2022-06-29

**Authors:** C Camacho, EM Camacho, DM Lee

**Affiliations:** Division of Population Health, Health Services Research & Primary Care, The University of Manchester, Manchester M13 9PL, UK; Division of Population Health, Health Services Research & Primary Care, The University of Manchester, Manchester, UK; Faculty of Health, Psychology and Social Care, Manchester Metropolitan University, Manchester, UK

**Keywords:** sexually transmitted infections, older people, epidemiology

## Abstract

**Aims:**

We describe the epidemiology of sexually transmitted infections (STIs) and HIV in people aged ⩾45 years in England and provide future projections about the burden of STIs in this age group.

**Methods:**

Analysis of national surveillance data in England from 2014 to 2019 for chlamydia, gonorrhoea, herpes, syphilis, anogenital warts and HIV was carried out. Time trends were assessed by the Poisson regression and reported using incidence rate ratios (IRRs). Two scenarios were modelled to predict the number of new STI diagnoses and associated costs in 2040.

**Results:**

In 2019, there were 37,692 new STI diagnoses in people ⩾45 years in England. Between 2014 and 2019, there was a significant increase in the rate of new STI diagnoses in men (IRR = 1.05, *p* = .05) and those aged 45–64 years (IRR = 1.04, *p* = .05). Absolute numbers of new STI diagnoses in men who have sex with men increased by 76% between 2014 and 2019 (IRR = 1.15, *p* < .001). In adults aged ⩾50 years, the number of episodes of care for HIV increased over time (age = 50–64 years, IRR = 1.10; age = 65+ years, IRR = 1.13; *p* <.001). The modelled scenarios predicted an increase in STI diagnoses and costs in older people by 2040.

**Conclusion:**

STI rates in England are increasing in people aged ⩾45 years. The population is ageing and older people will contribute an increasing burden to STI costs if this trend continues. The reasons for this trend are not fully understood and further longitudinal epidemiological research is needed. Sexual health promotion campaigns and healthcare interventions targeted at older people should be prioritised.

## Introduction

There is a broad consensus that the incidence of sexual health infections in older people (aged ⩾45 years) has increased over the last two decades.^1–3^ In 2015, the Annual Report of the Chief Medical Officer for England focused on the health of the ‘Baby Boomer’ generation, then aged 50–70 years. This reported that sexually transmitted infections (STIs) had increased by a third in this age group, with a notable increase in new HIV diagnoses and the proportion of older people living with HIV.^1^ Similarly, a recent international review found evidence of increasing STI rates in older people (typically ⩾50 years) in the US, Canada and Australia.^2^ A study using data from genitourinary medicine (GUM) clinics in one region of England showed that new STI diagnoses in people aged 45 years and over more than doubled between 1996 and 2003.^3^ The emerging recognition that the burden of STIs is increasing in older people is reflected in current health policy in England, with the National Institute for Health and Care Excellence (NICE) recently introducing the recommendation that healthcare professionals should ask older people about their sexual history and identify their risk of STIs.^4^

In stark contrast to this, the WHO’s 2021 report on global progress on STIs did not even report prevalence rates of STIs for people aged over 49 years.^5^ Recent evidence suggests that older people are hidden or marginalised in the area of sexual health and that they face significant barriers in seeking advice and treatment including stigma and embarrassment both on the part of older people and healthcare professionals.^6–8^ Contrary to popular assumptions, many older people remain sexually active and those having condomless sex with a new partner or multiple partners are potentially at risk of STIs.^2,9–11^ However, it remains unclear what factors are driving the increasing STI rates in older people, with physiological, behavioural and social theories being proposed.^11^

Like many high-income countries, England has an ageing population, with those aged over 45 years forecast to grow by 4.4 million people between 2018 and 2038.^12^ There is a growing body of data on sexual activity in older people, including those who remain sexually active into their 70s and 80s, with regular sexual expression associated with various positive health outcomes.^9,13^ Therefore, it is increasingly important to understand the evolving epidemiology of STIs in older adults so that behavioural factors, service delivery, and clinical and public health interventions can be ready to respond.

This article has the following two key aims:

1. To describe the epidemiology of STIs and HIV in people aged ⩾45 years in England between 2014 and 2019.
2. To estimate the future burden of STIs in this age group in England.

## Methods

This is a cross-sectional analysis of national surveillance STI data in England from 2014 to 2019.

### Data sources

The National Institute for Health Protection (NIHP) – formerly Public Health England (PHE) – has compiled data on STIs in England using information submitted by specialist and non-specialist^i^ sexual health services to the Genitourinary Medicine Clinic Activity Dataset Version 2 (GUMCADv2) Surveillance System. Data on chlamydia diagnosis are collated through the Chlamydia Testing Activity Dataset (CTAD) Surveillance System, also managed by PHE. Aggregate anonymised data from GUMCADv2 and CTAD were used for the STI analysis.^14,15^ HIV data collated by PHE from the HIV & AIDS New Diagnoses & Deaths Database and the HIV and AIDS Reporting System (HARS) were used at an aggregated level for the HIV analysis.^16^ Office of National Statistics (ONS) population estimates for 2014–2019 and projections for 2038 were used to calculate rates per 100,000 population.^17^

### Inclusion criteria

‘Older people’ is used to refer to people aged 45 years and over for STI analysis and 50 years and over for the HIV analysis (due to how the source data were aggregated by age). New STI diagnoses for named infections were available for chlamydia, gonorrhoea, herpes, syphilis, anogenital warts and HIV. A total new STI diagnosis count was available which also included *Mycoplasma genitalium* and *Shigella* infections from 2015 onwards. Analysis of trends over time for all total new STIs excluded data from 2014 due to the change in definition for this measure. Information on age at HIV diagnosis, the number of people receiving HIV care and HIV late presentations by age were included. HIV late presentations are defined at people with a CD4 count below 350 cells/μL at diagnosis.^18^ HIV was excluded from modelling as this is an established discipline with recommended methodologies which were outside the scope of this article.^19^

### Data analysis

The unit of analysis is a new episode of infection so individuals may be duplicated in the data set if they have been diagnosed with more than one STI. The rates of new infections per 100,000 population were calculated using the ONS population estimates.^17^ Time trends were assessed by the Poisson regression and are reported using incidence rate ratios (IRRs) and their 95% confidence intervals (CIs). Data were analysed using Stata v17 (Stata Corp, College Station, TX, USA). Two scenarios which predict the number of new diagnoses of chlamydia, gonorrhoea, herpes, syphilis and warts in older people in 2040 were modelled. The first model (demographic change model) assumes that the rate of new STI diagnoses in people aged over 45 years will remain the same as it is in 2019; changes in the number of STI diagnoses are due to changes in population structure (i.e. the projected number of people aged over 45 years in England in 2040). The second model (continuing trend model) assumes that the rate of STI diagnoses in people aged over 45 years will continue to change at the rate observed between 2014 and 2019.

### Economic modelling

Current National Health Service (NHS) recommendations for the treatment of STIs (excluding HIV) were used to estimate the associated costs (from an NHS perspective) using a bottom-up approach.^20^ As HIV is a lifelong condition, rather than modelling costs for new cases, the number of episodes of secondary care was used as a measure of resource use. NHS unit costs were derived from the most recent versions of the NHS reference costs database,^21^ Personal Social Services Research Unit (PSSRU) unit costs of health and social care,^22^ and the NHS drug tariff.^23^ Unit costs included in the analysis are reported in Supplemental Appendix 1. The unit cost (per case/episode) was multiplied by the predicted number of cases/episodes for each model scenario.

### Ethics and patient and public involvement

No ethical approval was required for this secondary analysis of PHE data. There was no direct patient or public involvement in this analysis.

## Results

In 2019, there were 468,342 new STI diagnoses in England, of which 37,692 (8.0%; 95% CI = 8.0%–8.1%) occurred in people aged 45 years and over. In people aged ⩾45 years, chlamydia was the most common diagnosis comprising 26.1% of the total (*n* = 9849), followed by gonorrhoea with 19.3% (*n* = 7263). The majority of new diagnoses were in males (73.7%, *n* = 27,786) and people aged 45–64 years (92.6%, *n* = 34,921). The most common route of transmission was between men who have sex with men (MSM), who accounted for 36.7% (*n* = 13,834) of new diagnoses in 2019 (Table 1).

**Table 1:** New STI diagnoses for people aged 45 years and over; new HIV diagnoses, episodes of care and late diagnoses for people aged 50 years and over in England by diagnosis, gender and age group.

	2014	2015	2016	2017	2018	2019	IRR (95% CI)	*p*-value
Diagnosis								
Chlamydia	5870	6042	6343	6890	8534	9849	1.10 (1.01–1.20)	0.03
Gonorrhoea	3317	3894	3602	4461	6061	7263	1.16 (1.05–1.30)	0.01
Herpes	4134	4119	4095	4027	4197	4185	0.99 (0.88–1.11)	0.87
Syphilis	1062	1279	1573	1896	1995	2169	1.14 (0.95–1.36)	0.17
Warts	5441	5374	5163	5129	5412	5317	0.98 (0.89–1.09)	0.76
All new STIs^a^	30,902^b^	31,233	30,878	31,895	35,544	37,692	1.04 (0.99–1.10)	0.13
Gender								
M	22,028^b^	22,489	22,079	23,032	26,051	27,786	1.05 (1.00–1.09)	0.03
F	8855^b^	8709	8724	8788	9309	9750	1.02 (0.95–1.10)	0.63
Age group								
45–64	28,803^b^	29,096	28,776	29,602	32,922	34,921	1.04 (1.00–1.09)	0.05
65+	2099^b^	2137	2102	2293	2622	2771	1.06 (0.93–1.21)	0.36
Route of transmission (excludes HIV)								
Male (heterosexual)	12,481^b^	12,275	12,057	11,532	11,511	11,190	0.98 (0.97–0.98)	<0.001
Male MSM	7880^b^	8448	8338	9691	11,868	13,834	1.15 (1.14–1.16)	<0.001
Female (heterosexual)	7414^b^	7449	7413	7398	7663	7982	1.02 (1.01–1.02)	<0.001
Female WSW	32^b^	26	20	33	43	70	1.35 (1.21–1.50)	<0.001
HIV								
New HIV diagnoses	1051	903	885	802	845	850	0.94 (0.75–1.18)	0.61
New diagnoses by gender								
M	758	664	642	574	613	583	0.93 (0.78–1.12)	0.47
F	293	239	243	227	232	267	0.96 (0.71–1.31)	0.82
New diagnoses by age group								
50–64	847	758	738	684	711	714	0.95 (0.80–1.13)	0.57
65+	204	145	147	118	134	136	0.91 (0.62–1.34)	0.63
HIV episodes of care								
50–64	19,700	22,181	24,784	27,223	29,509	32,275	1.10 (1.10–1.10)	<0.001
65+	3180	3613	4100	4639	5252	5985	1.13 (1.13–1.14)	<0.001
HIV late diagnoses								
50–64	342	292	279	229	272	258	0.95 (0.92–0.97)	<0.001
65+	67	58	64	47	47	44	0.92 (0.86–0.98)	0.01

STI: sexually transmitted infection; HIV: human immunodeficiency virus; IRR: incidence rate ratio; CI: confidence interval; MSM: men who have sex with men; WSW: women who have sex with women; M: male; F: female.

a Includes other STIs so total different.

b Not included in regression model.

Analysis of all new STI diagnoses (including HIV) at the local authority level showed an uneven geographical distribution of STIs in adults aged ⩾45 years. Out of 150 English local authorities, 38 had an STI rate higher than 160 per 100,000 population over 45 years. This included 26 out of the 33 London boroughs, with Brighton and Hove, Manchester and Southampton having the highest rates outside of London (Supplemental Appendix 2).

### Trends over time

Between 2014 and 2019, there were 198,144 new STI diagnoses in people aged 45 years and over (Table 2). Over this time period, there was a significant increase in the rate of new gonorrhoea diagnoses (IRR = 1.16, *p* = .01) and chlamydia diagnoses (IRR = 1.10, *p* = .03). The majority of new STI diagnoses were in men (*n* = 143,465, 73.7%). People in the 45–64 years age group accounted for 92.6% of STI diagnoses in those aged over 45 years (*n* = 184,120). The most common route of transmission in 2014 was in heterosexual men, who accounted for 40.4% of new STI diagnoses (*n* = 12,481). Over time, this has changed, and the dominant route of transmission in 2019 was among MSM (*n* = 13,843, 36.7%), which increased by 76% between 2014 and 2019 (IRR = 1.15, *p* < .001).

**Table 2:** Trends over age, gender and time for new STI diagnoses.

Age (years)	2014	2015	2016	2017	2018	2019	IRR (95% CI)	*p*-value
Males								
13–14	120	108	96	101	88	90	0.93 (0.80–1.09)	0.38
15–19	25,495	23,466	21,323	20,970	21,350	21,702	0.99 (0.98–1.01)	0.53
20–24	73,133	69,556	65,228	64,536	66,808	67,444	1.00 (0.99–1.01)	0.77
25–34	82,192	83,245	79,548	80,233	87,091	94,029	1.03 (1.02–1.04)	<0.001
35–44	31,271	31,698	29,808	30,532	34,126	38,353	1.05 (1.03–1.08)	<0.001
45–64	20,314	20,771	20,367	21,176	23,943	25,584	1.05 (1.02–1.09)	<0.001
65+	1714	1718	1712	1856	2108	2202	1.05 (0.96–1.16)	0.28
Females								
13–14	1070	806	615	605	610	586	0.91 (0.86–0.97)	<0.001
15–19	62,207	55,796	52,312	50,893	50,063	49,389	0.98 (0.97–0.99)	<0.001
20–24	79,061	74,543	73,198	73,747	76,702	79,843	1.03 (1.02–1.04)	<0.001
25–34	52,691	52,479	52,503	53,507	56,575	59,213	1.03 (1.01–1.05)	<0.001
35–44	15,125	14,736	14,962	15,069	15,905	16,891	1.03 (1.00–1.06)	0.04
45–64	8471	8307	8351	8354	8837	9208	1.02 (0.96–1.08)	0.55
65+	384	402	373	434	472	542	1.07 (0.86–1.34)	0.52
Persons								
13–14	1202	921	723	710	708	678	0.91 (0.84–0.99)	0.02
15–19	88,259	79,824	74,316	72,358	71,951	71,649	0.99 (0.97–1.00)	0.02
20–24	152,745	144,698	139,357	138,947	144,175	148,107	1.02 (1.01–1.03)	<0.001
25–34	135,042	136,020	132,560	134,131	144,102	153,750	1.03 (1.02–1.05)	<0.001
35–44	46,431	46,478	44,902	45,719	50,170	55,400	1.05 (1.02–1.07)	<0.001
45–64	28,803	29,096	28,776	29,602	32,922	34,921	1.04 (1.00–1.09)	0.05
65+	2099	2137	2102	2293	2622	2771	1.06 (0.93–1.21)	0.36

STI: sexually transmitted infection; IRR: incidence rate ratio; CI: confidence interval.

### Trends over age and time

Younger people continue to bear the highest burden of STIs; between 2014 and 2019, people aged under 45 years accounted for 92.5% of new STI diagnoses (*n* = 2,456,033). However, in the youngest age groups, there is a significant downwards trend in the rate of new STI diagnoses while in the older age groups, including those aged 45–64 years, the rates of STI are increasing (Table 2).

### HIV

In 2019, there were 850 new HIV diagnoses in people aged 50 years and over in England (Table 1). This decreased from 1051 new diagnoses in 2014, although the trend failed to reach significance (IRR = 0.94, *p* = .61). Between 2014 and 2019, the over 50s population increased by 1.7 million people, from 19.4 to 21.0 million. In this period, there were 5336 new HIV diagnoses in older people, 72% (*n* = 3834) of which were in men (vs women). The ratio of male-to-female diagnoses remained stable at 2.5:1. There were no significant trends over time for new HIV diagnoses in adults aged ⩾50 years.

In 2019, there were 38,260 episodes of HIV care for older people in England, which represents 42.4% of all HIV care episodes (Table 3). This is an increase of 13.1 percentage points in the proportion of adults aged ⩾50 years receiving HIV care from 2014. Episodes of HIV care increased significantly in both the 50–64 years (IRR = 1.10, *p* < .001) and the over 65 years age groups (IRR = 1.13, *p* < .001). Less than 2000 older adults received a late HIV diagnosis between 2014 and 2019. There was a significant decreasing trend for late diagnosis in adults aged 50–64 years (IRR = 0.95, *p* = .001) and those aged over 65 years (IRR = 0.92, *p* = .01).

**Table 3:** Trends and costs over age, gender and time for HIV episodes of care (EoC).

Age (years)	2014		2019		Trend from 2014 to 2019		
	EoC	Cost^a^	EoC	Cost^a^	IRR (95% CI)	*p*-value	Cost change
Male							
<15	221	£61,438	110	£30,580	0.87 (0.84–0.91)	<0.001	−£30,858
15–24	1606	£446,468	1345	£373,910	0.95 (0.94–0.97)	<0.001	−£72,558
25–34	8224	£2,286,272	8426	£2,342,428	1.00 (1.00–1.01)	0.64	£56,156
35–49	25,140	£6,988,920	23,989	£6,668,942	0.99 (0.99–0.99)	<0.001	−£319,978
50–64	14,827	£4,121,906	23,283	£6,472,674	1.09 (1.09–1.10)	<0.001	£2,350,768
65+	2613	£726,414	4740	£1,317,720	1.12 (1.12–1.13)	<0.001	£591,306
Total	52,631	£14,631,418	61,893	£17,206,254	1.03 (1.03–1.03)	<0.001	£2,574,836
50+	17,440	£4,848,320	28,023	£7,790,394	1.10 (1.09–1.10)	<0.001	£2,942,074
Female							
<15	259	£72,002	153	£42,534	0.91 (0.88–0.94)	<0.001	−£29,468
15–24	907	£252,146	788	£219,064	0.97 (0.96–0.99)	<0.001	−£33,082
25–34	4347	£1,208,466	2817	£783,126	0.91 (0.91–0.92)	<0.001	−£425,340
35–49	14,355	£3,990,690	14,284	£3,970,952	1.00 (0.99–1.00)	0.25	−£19,738
50–64	4872	£1,354,416	8992	£2,499,776	1.13 (1.12–1.14)	<0.001	£1,145,360
65+	567	£157,626	1245	£346,110	1.17 (1.15–1.19)	<0.001	£188,484
Total	25,307	£7,035,346	28,279	£7,861,562	1.02 (1.02–1.02)	<0.001	£826,216
50+	5439	£1,512,042	10,237	£2,845,886	1.13 (1.13–1.14)	<0.001	£1,333,844
Persons^b^							
<15	482	£133,996	263	£73,114	0.89 (0.87–0.92)	<0.001	−£60,882
15–24	2516	£699,448	2133	£592,974	0.96 (0.95–0.97)	<0.001	−£106,474
25–34	12,572	£3,495,016	11,243	£3,125,554	0.98 (0.97–0.98)	<0.001	−£369,462
35–49	39,499	£10,980,722	38,273	£10,639,894	0.99 (0.99–0.99)	<0.001	−£340,828
50–64	19,700	£5,476,600	32,275	£8,972,450	1.10 (1.10–1.10)	<0.001	£3,495,850
65+	3180	£884,040	5985	£1,663,830	1.13 (1.13–1.14)	<0.001	£779,790
Total	77,949	£21,669,822	90,172	£25,067,816	1.03 (1.03–1.03)	<0.001	£3,397,994
50+	22,880	£6,360,640	38,260	£10,636,280	1.11 (1.10–1.11)	<0.001	£4,275,640

HIV: human immunodeficiency virus; EoC: episode of care; IRR: incidence rate ratio; CI: confidence interval.

a Based on 2019 NHS reference cost – outpatient HIV (stable patients) £278 per episode.

b Including where gender is ‘unknown’ or ‘other’.

### HIV episodes of care – trends and costs over age and time

There has been a downwards trend over time in HIV episodes of care (EoC) for those in younger age groups, while in adults aged 50 years and over, the number of EoC has increased over time (age = 50+ years, IRR = 1.11, *p* < .001), which partly reflects the changing demographic of people living with HIV over time.In terms of NHS costs for HIV care, there was a net increase of £3.4m between 2014 and 2019. This was driven by the over 50 years age group whose costs increased by £4.3m, whereas in younger age groups, costs decreased by £0.88m in this period (Table 3).

### Activity and cost projections

The total number of diagnoses for chlamydia, gonorrhoea, herpes, syphilis and warts in 2019 among older people in England was 28,660. The cost of treating these STIs in 2019 was estimated to be £2.97m. Both scenarios projected an increase in STI diagnoses in people aged over 45 years by 2040 (Table 4). By 2040, annual NHS treatment costs for STIs in people aged 45 years and over are estimated to be between £3.1m (assuming STI rates remain the same but reflecting demographic population change) and £40.5m (assuming the changes in STI rates observed between 2014 and 2019 continue).

**Table 4:** Cases of STIs and estimated treatment costs for 2019, with projections for 2040.

	2019	Model 1 – 2040 (95% CI)	Model 2 – 2040 (95% CI)
Actual and projected new STI diagnoses			
Chlamydia	9788	10,156 (9,958–10,353)	95,169 (94,565–95,774)
Gonorrhoea	7241	7521 (7351–7691)	192,609 (191,749–193,469)
Herpes	4174	4376 (4246–4506)	4283 (4155–4412)
Syphilis	2163	2259 (2165–2352)	36,085 (35,713–36,458)
Warts	5294	5577 (5431–5723)	4483 (4352–4614)
Total	28,660	29,888 (29,151–30,624)	332,630 (330,534–334,727)
Estimated and projected costs (£)			
Chlamydia	1,163,402	1,207,090 (1,183,612–1,230,567)	11,311,841 (11,239,973–11,383,710)
Gonorrhoea	909,325	944,442 (923,097–965,788)	24,187,850 (24,079,826–24,295,872)
Herpes	150,014	157,268 (152,608–161,928)	153,947 (149,337–158,558)
Syphilis	263,929	275,595 (264,229–286,961)	4,403,146 (4,357,715–4,448,577)
Warts^a^	484,941	510,857 (497,449–524,264)	410,640 (398,619–422,661)
Total	2,971,610	3,095,252 (3,020,995–3,169,508)	40,467,424 (40,225,470–40,709,378)

STI: sexually transmitted infection; CI: confidence interval; Model 1: demographic change model; Model 2: continuing trend model.

Data for 2019 include only cases where both age and gender were known.

a 30% of cases resolve without treatment.

## Discussion

Between 2014 and 2019, there was a significant increase in the rate of new STI diagnoses in people aged 45–64 years, with new gonorrhoea and chlamydia diagnoses roughly doubling over the 6-year period. Specifically, 2019 saw 37,692 new STI diagnoses in England in people aged 45 years and over, which was 8% of the total STI diagnoses. MSM and the 45–64 years age group have seen the highest increases in new STI diagnoses. Episodes of HIV care have also significantly increased in people aged 50 years and over. The modelled scenarios predicted an increase in STI diagnoses and costs in older people by 2040.

### Strengths and limitations

This study used a large administrative data set which included all STI and HIV diagnoses in England in both specialist and non-specialist settings, thereby minimising bias. As far as we are aware, this is the first national analysis of the epidemiology of STIs in people aged over 45 years in England. We describe significant increases in STI rates in older people in England, consistent with global data from both high- and low-/middle-income countries showing increasing STI rates in older people.^24^ This study used data from England, but the trends and projections may be generalisable to other countries with similar demographic trajectories. A key limitation of this study relates to the source data being only available in aggregate form. This meant that individual level regression analysis could not be used to explore associations between socio-demographic factors and STI diagnoses. Data were aggregated either by diagnosis and age or diagnosis and ethnicity, so we were unable to include ethnicity in this analysis. There was also a change in the way STI data were collected in 2015 which meant that 2014 data could not be used when analysing trends for all new STIs. Finally, we did not use transmission dynamic modelling which is more accurate than epidemiological models in predicting future trends in new STI diagnoses.^25^ This was due to a lack of data on additional factors which may be associated with changing STI rates in older people, for example, behavioural data and social networks.^26^

### Context

Only one comparable study was identified, from the West Midlands STI Surveillance Project.^3^ The West Midlands is a region in England with a population of 5.9 million people. The rates of chlamydia, genital herpes, gonorrhoea and syphilis in people aged 45 years and over attending GUM clinical were analysed over an 8-year period from 1996 to 2003. The analysis included 4445 STI episodes in older people and found an overall doubling in STI rates over the study period.

Our analysis is broader in scope, in terms of geography, diagnoses and settings. Data cover the whole of England, with over 155,000 new STI diagnoses in older people, HIV is included, and activity data come from both specialist and non-specialist settings. Older people are less likely to attend a sexual health clinic after having condomless first sex with a new partner and/or ⩾2 partners and no condom use,^10^ so this analysis may be more representative including older people presenting, for example, to primary care. The West Midlands analysis used rate ratios, comparing their first and last data points on the number of episodes to assess temporal trends. We used incident rate ratios from the Poisson regression to assess trend, which uses data from all available years and takes into account the population at risk in each year. Our method found more modest changes in STI rates, but we believe it is more robust, and would allow comparison with future analyses looking at changes over different periods of time. The West Midland analysis did collect disaggregated data allowing a more in-depth data analysis describing activity in four narrower age bands, whereas we were limited to two broader groups. However, despite having ethnicity data, they reported they were unable to use this in their analysis due to small numbers.

The results of this study are consistent with the emerging global literature of STIs in older people. Surveillance data from the US, Canada and Australia all show increases in diagnosis rates of STIs in older people and older people living with HIV/AIDS.^24,27,28^ Older people are often excluded from epidemiological analysis of STI trends so there is a dearth of comparable studies.^5,29^

### STI risk factors for older adults

A range of physiological, socio-behavioural and structural factors have been proposed as explanations for the increasing STI incidence among older adults.^30^ Ageing is associated with reductions in immune response which could increase susceptibility to infection.^31^ Older women may be at an increased risk of acquired STIs due to reduced vaginal lubrication and thinning of the vaginal mucosa.^27^ Decreasing testosterone levels in men can lead to erectile problems which make condom use more difficult, contributing to increased STI risk.^27^ The widespread use of Sildenafil (Viagra) has allowed men to engage in penetrative sex later in life.^32^ Consistent with research in younger men, older MSM who report Viagra use are more likely to engage in unprotected sex.^33^

Social changes in recent decades, such as higher divorce rates, changing social attitudes and increasing foreign travel, have led to people taking new partners in later life, intergenerational relationships and engagement with sex workers.^11^ Older people are less likely to have had sex education at school, and are typically excluded from sexual health promotion programmes more often targeted at young people.^34^ These factors may lead to people being less able to negotiate safe sex in later life.^35^

Older people are less likely to seek help for STIs, have decreased condom use, lower rates of STI testing and delayed presentation for treatment.^10,36^ In a US sample of people aged 40–80 years, over 75% of those who experienced a sexual health problem did not seek help from a health professional.^37^ Healthcare professionals are less likely to initiate sexual health conversations with older people; the mutual reluctance from older people and healthcare professionals to raise sexual health concerns in a clinical setting present significant barriers to treatment.^38^

### Implications

The estimated annual treatment costs for cases of STIs in people aged over 45 years in 2019 were around £3m. The modelled scenarios for 2040 suggest that costs could increase modestly due to demographic changes (if STI rates remain as they are in 2019) or increase by more than 10-fold (if STI rates in this age group continue to change at the same rate as between 2014 and 2019). These estimates may be considered conservative and worst-case scenarios, respectively, but provide reasonable bounds of future healthcare spending in this area upon which commissioners can draw when planning sexual health services to meet the changing demands of the future as our population ages.

Policy-makers should continue to promote older people being asked about their sexual history in primary care settings^4^ and also consider the sexual health of this age group in future public health campaigns. A key area for further research is longitudinal studies of sexual behaviour and evaluation of health delivery and prevention interventions targeted at older people. Also, there are very limited data on the relationship between ethnicity and STIs in older adults. We have made assumptions about trends in STI rates using population-based projections, future analyses could use transmission dynamic modelling using behavioural parameters specific to older people.

## Conclusion

STI rates in England are increasing in people aged over 45 years. Although the numbers are small relative to younger people, this population demographic is increasing and people aged 45 years and over will contribute an increasing burden to STI costs if this trend continues. A combination of changing societal norms, structural barriers in accessing health care and knowledge about STIs and increased biological susceptibility are all likely contributors to these increasing STI rates. Further epidemiological research is needed to assess trends and causal associations. This article highlights a clear need for sexual health promotion campaigns and healthcare interventions targeted at people aged over 45 years.

## Supplemental Material

sj-pdf-1-rsh-10.1177_17579139221106348 – Supplemental material for Trends and projections in sexually transmitted infections in people aged 45 years and older in England: analysis of national surveillance data Click here for additional data file. Supplemental material, sj-pdf-1-rsh-10.1177_17579139221106348 for Trends and projections in sexually transmitted infections in people aged 45 years and older in England: analysis of national surveillance data by C Camacho, EM Camacho and DM Lee in Perspectives in Public Health

## Notes

i. Specialist (Level 3) services: genitourinary medicine (GUM) services and integrated GUM/SRH services. Non-specialist (Level 1 and Level 2) services: SRH services, young people’s services, online sexual health services, termination of pregnancy services, pharmacies, outreach and general practice, and other community-based settings.
