# Supplementary material for: Trends and projections in sexually transmitted infections in people aged 45 years and older in England: analysis of national surveillance data
Source: Perspect Public Health. 2022 Jun 29;143(5):263–71. doi: 10.1177/17579139221106348 (PMC10576406; doi:10.1177/17579139221106348)
Supplement: sj-pdf-1-rsh-10.1177_17579139221106348 – Supplemental material for Trends and projections in sexually transmitted infections in people aged 45 years and older in England: analysis of national surveillance data [file sj-pdf-1-rsh-10.1177_17579139221106348.pdf]

Trends and projections in sexually transmitted infections in people aged 45 and older in England: analysis of national surveillance data

Supplementary Data

Contents

Appendix 1 - Description of STI treatment costs 1

Appendix 2 - The rate and number of new STI diagnoses in older people in local authorities with a rate ≥160/100,000 population 2

Appendix 1 - Description of STI treatment costs

STI	Resources	Unit cost
HIV	Outpatient visit for stable HIV patient	£278[1]
Chlamydia	Visit to GUM clinic plus treatment with doxycycline, 100mg twice daily for 7 days	£118.86[1,2]
Gonorrhoea	Visit to GUM clinic plus treatment with Ceftriaxone 1g IM injection	£125.58[1,2]
Herpes	Visit to GP, oral acyclovir, 400mg three times per day for 5-10 day	£35.94[2,3]
Syphilis	Visit to GUM clinic plus treatment with intramuscular injection of benzylpenicillin	£122.02[1,2]
Warts	Visit to GUM clinic plus treatment with podophyllotoxin 0.5% solution<br><br>*30% of cases resolve without treatment	£130.86[1,2]

References

1. NHS England. *National Schedule of Reference Costs 2019/20*. NHS England, 2020.
2. NHS Business Services Authority. NHS Electronic Drug Tariff, https://www.drugtariff.nhsbsa.nhs.uk/#/00782291-DD/DD00782285/Home (2020, accessed 27 April 2022).
3. Curtis, Burns. Unit Costs of Health and Social Care 2020. *Pers Soc Serv Res Unit Univ Kent Canterb*.

Appendix 2 - The rate and number of new STI diagnoses in older people in local authorities with a rate ≥160/100,000 population

Local Authority	PHE Centre	Number of new STI diagnoses (2018)	Rate of STI diagnoses per 100,000 (2018)
City of London	London	60	1522
Lambeth	London	1203	1276
Southwark	London	914	957
Hackney	London	573	777
Westminster	London	670	754
Islington	London	502	750
Kensington and Chelsea	London	447	697
Tower Hamlets	London	492	686
Camden	London	530	618
Lewisham	London	520	531
Brighton and Hove	South East	571	530
Hammersmith and Fulham	London	322	526
Wandsworth	London	478	506
Haringey	London	403	451
Greenwich	London	394	423
Brent	London	477	408
Manchester	North West	477	314
Waltham Forest	London	284	311
Newham	London	289	308
Croydon	London	442	294
Ealing	London	370	293
Southampton	South East	236	277
Merton	London	202	270
Portsmouth	South East	206	262
Blackpool	North West	175	260
Richmond upon Thames	London	200	238
Hounslow	London	207	218
Enfield	London	261	209
Bristol, City of	South West	316	208
Salford	North West	195	208
Reading	South East	112	201
Kingston upon Thames	London	127	193
Birmingham	West Midlands	701	180
Barking and Dagenham	London	114	178
Barnet	London	263	177
Luton	East of England	120	164
Hampshire	South East	1104	163
Bournemouth, Christchurch and Poole	South East	297	160
